# PCA-Based Denoising Algorithm for Outdoor Lidar Point Cloud Data

**DOI:** 10.3390/s21113703

**Published:** 2021-05-26

**Authors:** Dongyang Cheng, Dangjun Zhao, Junchao Zhang, Caisheng Wei, Di Tian

**Affiliations:** School of Aeronautics and Astronautics, Central South University, Changsha 410083, China; 195811030@csu.edu.cn (D.C.); junchaozhang@csu.edu.cn (J.Z.); caisheng_wei@csu.edu.cn (C.W.); 205811015@csu.edu.cn (D.T.)

**Keywords:** PCD filter, grid PCA, ground noise, KD-tree, normal vector, KNN

## Abstract

Due to the complexity of surrounding environments, lidar point cloud data (PCD) are often degraded by plane noise. In order to eliminate noise, this paper proposes a filtering scheme based on the grid principal component analysis (PCA) technique and the ground splicing method. The 3D PCD is first projected onto a desired 2D plane, within which the ground and wall data are well separated from the PCD via a prescribed index based on the statistics of points in all 2D mesh grids. Then, a KD-tree is constructed for the ground data, and rough segmentation in an unsupervised method is conducted to obtain the true ground data by using the normal vector as a distinctive feature. To improve the performance of noise removal, we propose an elaborate K nearest neighbor (KNN)-based segmentation method via an optimization strategy. Finally, the denoised data of the wall and ground are spliced for further 3D reconstruction. The experimental results show that the proposed method is efficient at noise removal and is superior to several traditional methods in terms of both denoising performance and run speed.

## 1. Introduction

With the development of three-dimensional (3D) technologies, lidar has gained increasing attention due to its low price, small size, and high precision. In recent years, lidars have been widely used in autonomous driving [1,2,3], aerospace [4], three-dimensional modeling [5,6,7,8], and other fields. In these applications, lidars provide the range map of the concerned object or environment, which can be further processed for geological mapping [9,10,11], simultaneous localization and mapping (SLAM) [12,13,14,15,16,17,18], as well as 3D modeling and reconstruction, even for the modeling of human organs, detection and positioning of necrotic tissues, and other aspects in medicine [19]. Generally, the 3D structure of the concerned object is scanned by lidar and restored after point cloud scan registration, segmentation, and reconstruction. Due to measurement errors, the raw point cloud data (PCD) are necessarily polluted by various noises [20], which potentially undermine the accuracy of 3D modeling. Consequently, denoising of PCD is the most important step in 3D point cloud processing before further applications.

In order to mitigate the impact of noise in PCD, researchers have developed many filters to remove noisy data [21]. Actually, we should usually compromise the efficiency and accuracy in the filter design, because more accurate filtering performance means more computational cost as well as more time-consuming, while a more efficient algorithm means more details of PCD have abandoned rendering loss of accuracy. Yao et al. [22] proposed a filtering method based on kernel density estimation (KDE), and the threshold was set according to the maximum probability density of points to accelerate the filtering process. Han et al. [23] deduced a linear model of guidance point cloud such that PCD can be filtered in a more efficient manner, which accompanied the decreased accuracy. In order to improve the filtering accuracy, Li et al. [24] proposed an improved adaptive filter to generate a filter and high-precision point cloud that can effectively reduce the error. In [25], PCD was first segmented before filtering to boost performance. How to effectively remove ground noise is always a troublesome problem in environmental 3D modeling. Therefore, many targeted filtering methods [26] exist to deal with this problem. Yuxiang et al. [27] extracted the digital terrain model by using the spatial structure of terrain and the spatial coherence between points thereby a good filter scheme for ground noise. Feichter et al. [28] identified a plane from a PCD, and generalized a description of shape features using triangulation of the boundary to remove noisy points. Liu et al. [29] proposed a surface modeling method based on the mixed regression technique to determine the plane. Quan et al. [30] proposed a method based on the filtering of adjacent triangular point clouds in an irregular triangulation network to effectively remove ground noise, therefore a noise-free ground model. However, this method will remove some useful features in PCD in some scenarios, hence some manual restrictions are required to prevent the loss of information. Leal et al. [31] proposed a filter based on the methods of median filtering and sparse regularization to preserve the sharp noise while removing noise in a PCD. In [32], the authors specially designed filter schemes for different regions of a PCD in order to farthest preserve the useful information in the PCD, while reducing the universality of the proposed filter. A more universal method based on spatial clustering was proposed by Xu and Yue [33] to remove noise while preserving complex ground information. Zou et al. [34] utilized the farthest point weighted mean down-sampling (FWD) technique to search the feature lost due to filtering operation and to retain feature points while denoising. Moreover, there exist other denoising filters based on deep learning [35,36], morphology [37], F-transform [38], etc.

**Figure 1:**
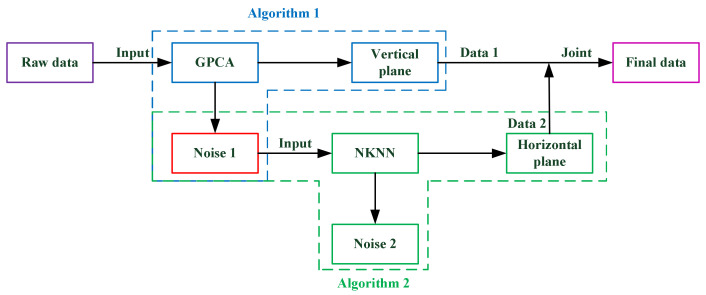
The general idea of the filtering method is presented. Algorithm 1 is used to process the initial data, and the wall point cloud and noise 1 are obtained. Noise 1 is composed of the ground point cloud and noise. Then, Algorithm 2 is used to process noise 1, and the ground point cloud and noise are separated. Finally, the wall and the ground are joined together.

When outdoor environmental modeling [39] is carried out with lidar, point cloud noise is generated at the stage of obtaining PCD. The aforementioned methods can effectively remove discrete outlier points which are treated as a noisy target. However, by using these methods, it is difficult to remove high-density noises, such as indoor environmental noises, which are relatively dense. This paper proposes a new filter scheme based on the distribution characteristics of indoor and outdoor point clouds for effectively handling high-density noise. The 3D point cloud is preprocessed by principal component analysis (PCA) [40] method therefore the reduced 2-dimensional point cloud toward the ground, consequently, the feature points of the wall are separated by a carefully designed grid threshold, which depends on the statistical difference between the pure ground points and the wall points projecting on the mesh grid of ground. This process is called grid principal component analysis (GPCA). Then, KD-tree [41] for the filtered point cloud is established to estimate normal vector features, which then facilitate the further plane segmentation. After the plane is segmented based on the angles between the normal vectors, the ground model is further optimized by the K nearest neighbor (KNN) method. The main idea of the proposed filter method is shown in Figure 1. Experimental comparisons with other traditional filters show that the proposed method is significantly advantageous over than other traditional methods, including statistical outlier removal (SOR) filter [42], voxel grid statistical outlier removal (VGSOR), radius outlier removal (ROR) filter [43], Gaussian filter [44], and difference of normals (DON) filter [45], for removing indoor point cloud noise.

In general, point density, which is an important parameter of lidar data [46], dramatically affects the performance of the denoising filter. Specifically, higher density means more difficult and computational cost in noise removal [47]. SOR filter is a simple method for eliminating noisy data by judging whether the average distance of the nearest points is within the threshold, hence it is sensitive to the sparse noisy points and incapable of high-density noises. To overcome this, down-sampling the data with voxel grids before discretizing the block noise is used in the VGSOR filter for lowering point density, therefore the SOR filter can be used. In this manner, the denoising performance for high-density PCD can be improved, however, some useful features in the point cloud are taken as noise since the down-sampling technique will render the loss of accuracy. In the ROR filter method, noises are determined by the specified number of points within a prescribed radius, and the outliers can be well handled by utilizing ROR, however, the computational cost and time consumptions of ROR are overlarge. The Gaussian filter is originally designed for image smoothing, while for PCD filtering, it manipulates the distances of the nearest points by a Gaussian distribution function, therefore the corresponding weight of each nearest point. Thus, one can take the weighted sum of the nearest points after normalization as a new point. In the DON filter, after time-consuming calculation of the included angle between the small range and large range normal vectors of each point, one can determine an appropriate threshold value for effectively eliminating noise.

The rest of this paper is organized as follows. Section 2.1 presents the principle of GPCA and its usage for PCD processing, then the normal-vector K nearest neighbor (NKNN) method is detailed in Section 2.2. In Section 3.1, the hardware equipment that we used in the experiment, as well as the data acquisition details, are briefly introduced. In Section 3.2, the proposed method is detailed step by step followed by a large number of experimental validations, as well as comparative studies in Section 3.3. Section 4 discusses the performance of the proposed method and concludes the advantages and disadvantages in some aspects, while the causes of defects are analyzed. At last, the conclusions and some future prospects are presented in Section 5.

## 2. Methods

In this section, we divide the proposed algorithm into two steps for detailed introduction: (1) by using the GPCA method, the wall, and noisy ground are accurately separated from the initial PCD; (2) by using NKNN, the normal vector segmentation, and optimization are carried out for separate ground from noisy PCD.

### 2.1. Filtering Based on GPCA

**Figure 2:**
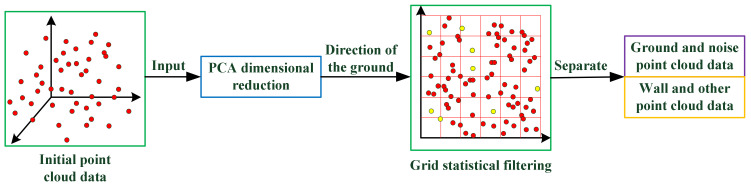
The filtering method based on grid principal component analysis (GPCA) is presented. The principal component analysis (PCA) algorithm is used to reduce the dimension of the initial data. A grid is constructed for the dimensionality reduction data. The number of points in the grid is counted. The data are divided into two parts by setting the threshold $k_{\sigma }$.

PCA is mainly used for reducing the dimensionality of complex data sets. As for 3D PCD, PCA can be used to obtain the normal vector of a point cloud plane. In order to reduce the complexity of 3D point cloud processing and to effectively remove noise, we firstly project a 3D point cloud onto a two-dimensional plane. Then, a statistical grid method is used to separate the ground and noise. The basic idea of GPCA is illustrated in Figure 2, and its implementing procedure is shown in Algorithm 1.

Firstly, dimensionality reduction via PCA is carried out for PCD. Assuming the input point cloud data $S\in {\mathbb{R}}^{n\times 3}$, the covariance matrix Γ of S is performed a singular value decomposition (SVD) decomposition, therefore the corresponding eigenvectors. The first two eigenvectors are selected as dimensionality reduction matrix for the input S as follows:

(1) $$S_{q}=S\Delta_{T}$$

where $\Delta_{T}\in {\mathbb{R}}^{3\times 2}$ transforms the 3D point set S into a 2D point set denoted by $S_{q}=\{x_{i},y_{i}\},i=1,\ldots ,n$, which represents the point set after dimensional reduction.

Then, we mesh the obtained two-dimensional data and set the number of mesh grids to be l. The mesh resolutions $b_{x}$ and $b_{y}$ respectively along the x and y directions are given by:

(2) $$\left\{\begin{array}{l} b_{x}=\frac{x_{\max}-x_{\min}}{l}\\ b_{y}=\frac{y_{\max}-y_{\min}}{l} \end{array}\right.$$

(3) $$\left\{\begin{array}{l} h_{xi}=round((x_{i}-x_{\min})/b_{x})\\ h_{yi}=round((y_{i}-y_{\min})/b_{y}) \end{array}\right.$$

where $\left(h_{xi},h_{yi}\right)$ are the coded number for each point and constrained within 1 to l as follows:

(4) $$h=\left\{\begin{array}{l} 1,\;\;\;\;\;\;h=0\\ l,\;\;\;\;\;\;h>l \end{array}\right.$$

The number of points projected via PCA into a grid (i,j) is denoted by $k_{ij}$. In order to divide the point cloud into two parts, a threshold parameter $k_{\sigma }$ is set as

(5) $$x_{ij}\in \left\{\begin{array}{l} S_{f},\;\;\;\;\;\;k_{ij}<k_{\sigma }\\ S_{w},\;\;\;\;\;\;k_{ij}\geq k_{\sigma } \end{array}\right.$$

where $x_{ij}$ represents the points in the grid (i,j), $S_{f}$ refers to the ground and noise points set, and $S_{w}$ refers to the wall points set. $S_{f}$ can be taken as the input of the subsequent algorithm for further processing.
**Algorithm 1.** GPCA outliers removed***Input:***S(n×3 points)***Output:*** $S_{f}(n_{f}\times 3\;points),S_{w}(n_{w}\times 3\;points)$ 1: $S=(x_{n},y_{n},z_{n})$ 2: $\mu =\left(\frac{1}{n}\sum_{i=1}^{n}x_{i},\frac{1}{n}\sum_{i=1}^{n}y_{i},\frac{1}{n}\sum_{i=1}^{n}z_{i}\right)$ 3: ***for***
each point
$p_{i}=(x_{i},y_{i},z_{i})\in S$
***do*** 4:   $p_{i}=p_{i}-\mu$ 5: ***end for*** 6: C=S 7: $\Gamma =\frac{1}{n}C^{T}C$ 8: $U\Sigma V^{T}=\Gamma$ 9: $V=\left(t_{1},t_{2},t_{3}\right)$10: $\Delta T=(t_{1},t_{2})$11: $S_{q}=S\Delta T$12: $b_{x}=\left(x_{\max}-x_{\min}\right)/l$13: $b_{y}=\left(y_{\max}-y_{\min}\right)/l$14: ***for***
each point$v_{i}=\left(x_{i},y_{i}\right)\in S_{q}$
***do***15:   $h_{xi}=round((x_{i}-x_{\min})/b_{x})$16:   $h_{yi}=round((y_{i}-y_{\min})/b_{y})$17:   $h_{i}=(h_{xi},h_{yi})$18: ***end for***19: ***for***
each grid (i,j) 
***do***20:   $Count\;the\;number\;k_{ij}\;of\;points\;in\;each\;cell$21:   ***if***
$k_{ij}<k_{\sigma }$
***then***22:     Noise f ← Points in grid (i,j)23:   ***else***24:     Wall points w ← Points in grid (i,j)25:   ***end if***26: ***end for***27: $Get\;S_{f}\;and\;S_{w}$

### 2.2. Filtering Based on NKNN

Since the obtained $S_{f}$ in the last subsection contain both the ground and the noisy points, it is necessary to separate the ground point cloud from $S_{f}$ for facilitating the final splicing of the ground and the wall in PCD. In this paper, the ground point cloud is segmented by the included angle between the normal vectors of the points, and the category is optimized and classified by the KNN algorithm.

In order to efficiently obtain characteristic information of the point cloud, KD-tree for $S_{f}$ is constructed first, and then the K nearest neighbor $p_{i}$ of each point in the tree are found out. Next, the minimum sum of the distance between a plane and its nearest neighbors is calculated, then we take the normal vector of the plane as the characteristic of the corresponding point. With the utilization of PCA, the normal vector of the PCD can be rapidly obtained as follows.

For each group of the K nearest neighbor, the mean of the nearest neighbors and the deviation error are given by:

(6) $$\mu_{j}=\frac{1}{k}\sum_{j=1}^{k}x_{j}$$

(7) $${\tilde{x}}_{j}=x_{j}-\mu_{j}$$

where $\mu_{j}$ is the mean of the nearest neighbors; ${\tilde{x}}_{j}$ is the difference between the point and the mean value $\mu_{j}$. The corresponding deviation matrix C is defined by:

(8) $$C=[{\tilde{x}}_{1},{\tilde{x}}_{2},\ldots ,{\tilde{x}}_{k}]$$

Performing an SVD decomposition for $CC^{T}$ yields:

(9) $$U\Sigma V^{T}=CC^{T}$$

Then the eigenvector relating to the smallest eigenvalue in U is taken as the normal vector $v_{i}$ of the corresponding point.

The resulted normal vector is used to extract the main components of the ground. Since the point set $S_{f}$ to be processed contains the ground points, noisy points, and a small number of wall points, the threshold angle between the normal vectors should be carefully prescribed as a key index for the ground plane segmentation. The main steps of ground segmentation are listed as follows:

• Pick a random point $v_{i}$ from the initial data set Ω. Compare the angle θ between the normal vectors of $v_{i}$ and all the other points $v_{j}$ as follows:

(10) $$\theta =\frac{v_{i}\cdot v_{j}}{\left||v_{i}||||v_{j}|\right|}$$

• Set the threshold δ. If the angle θ of a $v_{j}$ is less than δ, then $v_{j}$ is classified as $v_{i}$. Take the remaining points as the new initial data Ω, and repeat the previous step until all points are classified.

The above procedure is very efficient since only using the included angle of the normal vector as the key index for classification, however, it may lead to errors for some complex scenarios. To reduce the error of classification, an amendment method based on KNN is used to perform further corrections.

**Figure 3:**
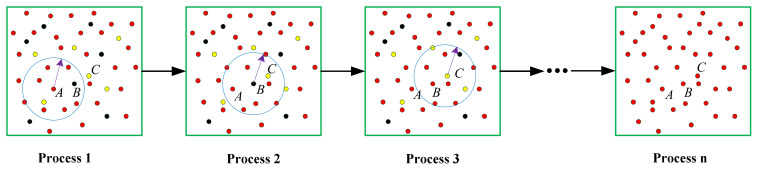
The classification results were corrected by K nearest neighbor (KNN). Each of these categories is represented by a different color. The radius is set as the starting point A, and a circle is drawn, as shown in Process 1. The categories within the statistical circle have the most dots in red. Therefore, the category A stays the same. In Process 2, it is found that the circle centered on B has more red dots; therefore, B is updated to red. In Process 3, C is updated to redpoint in the same manner. When each point is updated, points in the same plane are updated to the same class, as shown in Process n.

As shown in Figure 3, the KNN method was used to search the K nearest neighbor of the current point and to count the various categories of the nearest neighbor points. We took the category with the largest number as the category of the current point. Then, the class at that point was updated.

**Figure 4:**
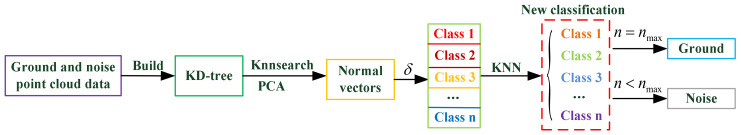
Filtering based on the normals K nearest neighbor (NKNN) is presented. The data obtained consists of ground and noise in the previous step. A KD-tree is built for the data. KD-tree is used to search for the KNN. Then, the PCA algorithm is used to quickly solve the normal vector. The data are classified by setting the threshold δ of the angle between the normal vectors. Then, the KNN algorithm is used for further optimization. Of all the categories, the category with the most points is the ground point cloud, and the others are noise.

Finally, the point corresponding to the largest class in the classified data was taken as the ground data. The data after filtering were obtained by splicing the ground and the wall in the second section. In the process of testing, it is necessary to properly adjust the four parameters of dimension reduction: the number l of grids, the threshold $k_{\sigma }$ of grid points, the included angle δ of the normal vector, and the number k of adjacent points. The purpose of this paper is to put forward a new method to solve the problem that point cloud noise in buildings cannot be removed by traditional filtering. The basic idea is shown in Figure 4 and Algorithm 2.
**Algorithm 2.** Filtering based on NKNN***Input:***$S_{f}(n_{f}\times 3\;points)$***Output:***$S_{o}(n_{o}\times 3\;points),S_{g}(n_{g}\times 3\;points)$ 1: $Construct\;the\;K-D\;tree\;of\;S_{f}$ 2: ***for***
each point
***do*** 3:   Calculate the k nearest neighbors 4:   $p_{i}=(x_{1},x_{2},\ldots ,x_{n})$ 5: ***end for*** 6: ***for***
neighbors of each point
***do*** 7:   $\mu_{j}=\frac{1}{k}\sum_{j=1}^{k}x_{j}$ 8:   ${\tilde{x}}_{j}=x_{j}-\mu_{j}$ 9:   $C=[{\tilde{x}}_{1},{\tilde{x}}_{2},\ldots ,{\tilde{x}}_{k}]$10:   $Compute\;SVD:\;U\Sigma V^{T}=CC^{T}$11:   $v_{i}\leftarrow The\;eigenvector\;of\;the\;minimum\;eigenvalue\;in\;U$12: ***end for***13: ***for***
$pick\;v_{i}\in \Omega \;at\;random$
***do***14:   ***for***
$each\;v_{j}\in \Omega$ do15:     $\theta =\frac{v_{i}v_{j}}{\left||v_{i}||||v_{j}|\right|}$16:     ***if***
θ<δ
***then***17:       $x_{i}\in Same\;class$18:       $remove\;v_{i}\;in\;\Omega$19:     ***end if***20:   ***end for***21:   ***if***
Ω is none
***then***22:     break23:   ***end if***24: ***end for***25: ***for***
$each\;point\;x_{i}$
***do***26:   nclass←the most class in the neighborhood27:   $x_{i}\in nclass$28: ***end for***29: ***if***
nclassismax
***then***30:   $Ground\;S_{g}\leftarrow the\;points\;of\;nclass$31: ***else***32:   $Noise\;S_{o}\leftarrow other\;nclass$33: ***end if***

## 3. Experimental Process and Results

In order to verify the effectiveness of the algorithm, the corresponding data were collected by lidar in this experiment. Finally, the algorithm was compared with traditional algorithms to evaluate its merits and demerits.

### 3.1. Hardware and Operating System

**Figure 5:**
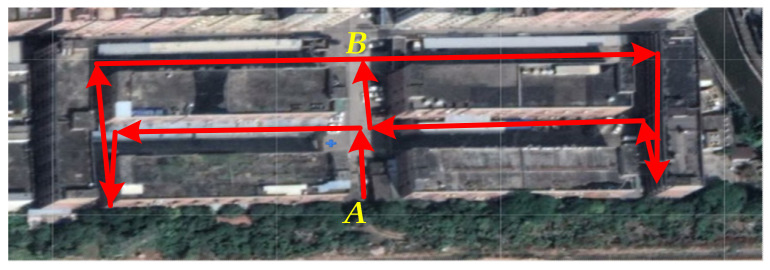
The experimental subject was a neighborhood. The red line is the path of movement when we measured the data. The *A* was the starting point from which we started the measurement, and the *B* was the endpoint.

**Figure 6:**
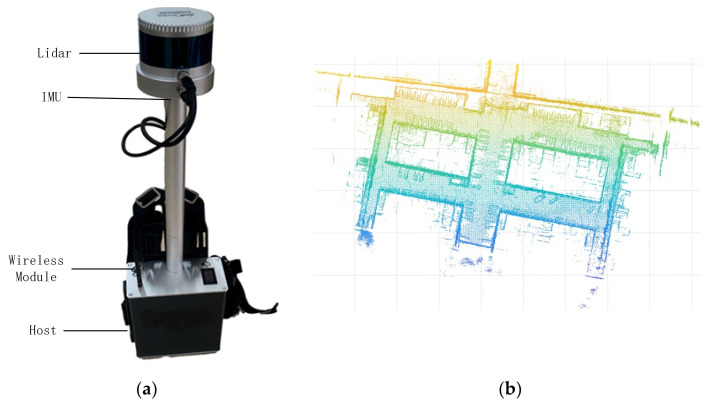
Backpack system and collected point cloud data (PCD): (**a**) the equipment we used to measure the data consists of a lidar, an inertial measurement unit (IMU), a wireless module, and a host. (**b**) After collection and scan registration, we obtained the three-dimensional data.

The experimental equipment was a backpack system integrating lidar, an inertial measurement unit (IMU), and an upper computer. The 16-line lidar was used to scan the environment and to return information about the position of the surface of the object. The IMU describes the attitude of the backpack system and feeds back information on the attitude change in the lidar. Each frame of the lidar and IMU data are matched using the robotic operating system (ROS) and fused into a complete point cloud. Finally, the upper computer uploads the PCD to a server through the wireless module and then transfers the data from the server to the client to achieve real-time monitoring. In this experiment, the system was used to model the community environment, as shown in Figure 5. The basic structure of the backpack is shown in Figure 6a, and about 170,000 points were obtained after system processing, as shown in Figure 6b.

During measurement, we moved along the red line to collect the data. In order to ensure the integrity of the data, we planned the route so that everything could be scanned by the lidar. We took the equipment in the middle of the road and moved about five meters away from the wall. Then, we carried backpacks and moved on foot along the path. The whole process lasted about three minutes. Finally, we got the complete data. The edge of the data is the surface of the building. The point clouds outside the edges of the figure, and inside the two rectangular edges in the middle are indoor debris measured by lidar through the glass of the building. The debris is the main PCD noise. Vehicles, trees, and other objects on the ground can be considered sub-essential cloud noise depending on the situation. Next, we took this data as an example to verify the algorithm.

### 3.2. Our Experiment Results in a One-by-One Step

**Figure 7:**
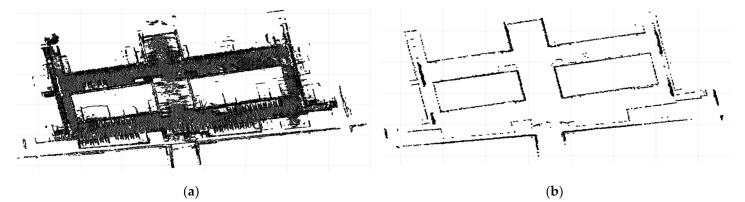
The process of the GPCA algorithm: (**a**) the initial 3D data are mapped to a 2D plane using the PCA algorithm and (**b**) the GPCA algorithm is used to filter the noise of the wall two-dimensional points. The features of the walls are well-preserved.

In order to verify the effectiveness of the algorithm, we evaluated the algorithm on indoor noise removal. In general, it was necessary to use a pass-through filter to crop the main PCD of the set for the initial data. Then, PCA was used to reduce the dimension of the point cloud. Generally, the first two columns of the dimensionality reduction matrix were selected to map the three-dimensional data to a two-dimensional plane that was close to the ground, as shown in Figure 7a. It can be seen that the number of information points on the ground does not change significantly. The obvious increase in the number of points is the regular wall mapping the two-dimensional formation of linear points. Therefore, a grid is used to divide the points after dimensionality reduction based on the difference in mapping points between the ground and wall. The number of meshes divided was determined according to the size of the point cloud obtained. Usually, the mesh can contain a part of the line segment of the wall map. In this experiment, the number of grids was set to 400 and the whole two-dimensional map was divided into 160,000 rectangular grids. Next, a threshold was set to eliminate all points in the grid where the number of points was less than the threshold. According to the statistics of the number of points in all the grids, the intersecting boundary of points in the grids is about 12. Therefore,12 was taken as the segmentation threshold in this experiment, and all grid points in the grid for which the number was less than 12 were separated to obtain the mapping points of the ground and noise. The wall was made up of the remaining points. Then, the corresponding 3D point cloud was restored through the label index of the point. The ground and noise were used for further processing. The wall was retained and spliced into a complete point cloud with the finally separated ground.

GPCA can effectively remove sparse noise points in Figure 7b. However, indoor noise tends to be as dense as or even denser than ground point clouds. If traditional filtering is used while increasing the intensity of filtering to remove high-density noise, some non-noise, such as part of the plane, is removed. By using GPCA, the high-density noise is removed by increasing the threshold value, and the point density of the wall is much higher than that of the ground and the noisy points after dimension reduction; therefore, the characteristics of the wall are better retained.

Since the ground point cloud and noise are eliminated simultaneously when GPCA is used, it is necessary to quickly extract the ground and to finish the wall splicing. In the remaining point clouds, only the ground has planarity and, thus, normal vector segmentation is used to quickly find the largest plane in the point cloud. In the algorithm, a SOR filter is used for data optimization. Then, a KD-tree is constructed on the point cloud to find the nearest neighbor points faster, and PCA is used to quickly fit the plane to obtain the normal vector of the corresponding point. In order to reduce the influence of edge noise when using a vector to segment the ground, the number of adjacent points used in this experiment was less than 20, and the included angle threshold of the normal vector was also smaller than 15 degrees. The results obtained only through normal vector segmentation are not ideal. The segmentation speed is fast, but the segmentation effect is very rough and even produces the phenomenon of overfitting. In order to improve the segmentation accuracy, the K nearest neighbor method was introduced as a constraint for further segmentation. We noted the category of the K nearest neighbor to the point, updated the category of the point to the category with the most points, and updated the next point. Thus, progressive point cloud fine segmentation was realized.

**Figure 8:**
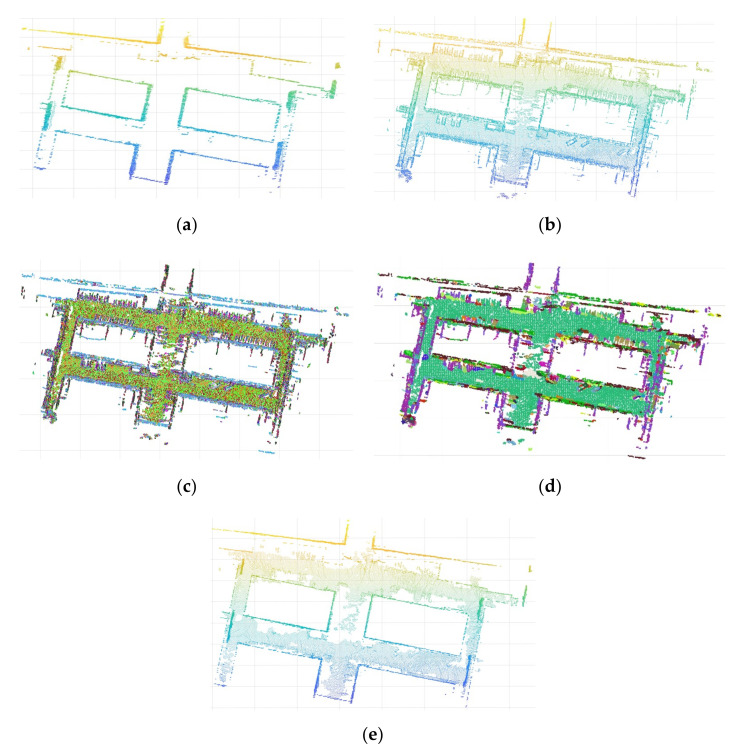
Segmentation and splicing of ground point clouds: (**a**) the two-dimensional wall is restored to three-dimensional point cloud data. (**b**) Other point clouds beside the wall: these point clouds are made up of ground and noise. (**c**) The data in (**b**) are roughly segmented by using the included angle of the normal vector. Point clouds on the ground are mostly grouped into the same category (the same color). However, many misclassified points still exist. (**d**) The classification results of the previous step are further segmented by the KNN principle, and the complete plane point cloud is obtained. (**e**) The ground and wall point clouds are joined together.

Before processing the noise, a SOR filter was used for preprocessing to facilitate segmentation and to achieve better results. The processed data are shown in Figure 8a,b. Figure 8a represents the wall point cloud after using GPCA, and Figure 8b represents point clouds other than the wall. Then, the initial segmentation points and the remaining points were randomly selected to calculate the included angle of the normal vector, and those less than the threshold value were classified into one category. The same was performed for the remaining points until all of the points were sorted. This is an unsupervised segmentation, and the number of categories obtained depends on the size of the angle between the normal vector set. In the end, we divided the noise into nine categories, and the results are shown in Figure 8c. It can be seen that the classification effect is not ideal, that the ground is roughly divided into two categories, and that the distribution of categories is disorderly. However, it can be found from the figure that the points occupying most of the ground are a class; therefore, the K nearest neighbor constraint was considered to centralize the class. It was also necessary to construct a KD-tree for the noise point cloud to quickly obtain the nearest neighbor points. The current point was updated based on the maximum category of the nearest neighbor points, and the updated effect is shown in Figure 8d. It can be seen that most points on the ground were classified into one category at this time, while the ground points corresponded to the category with the largest number of points after classification. This category was used as ground points and wall point clouds joined together to obtain the final point cloud, shown in Figure 8e.

### 3.3. Comparison with Other Methods

**Figure 9:**
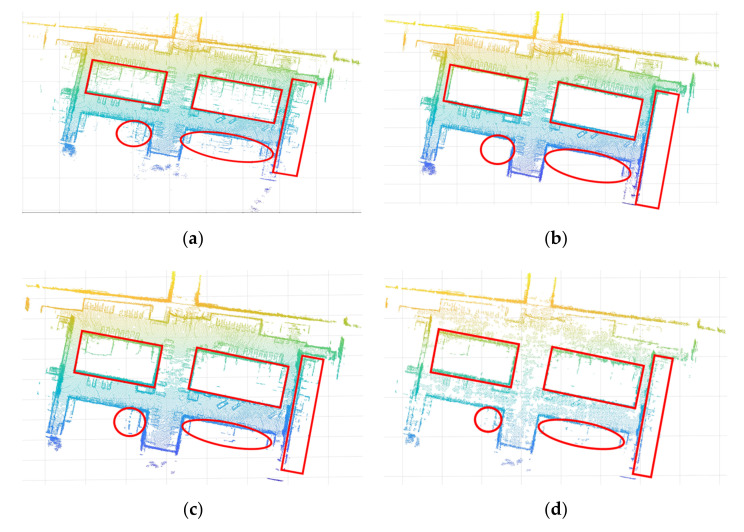

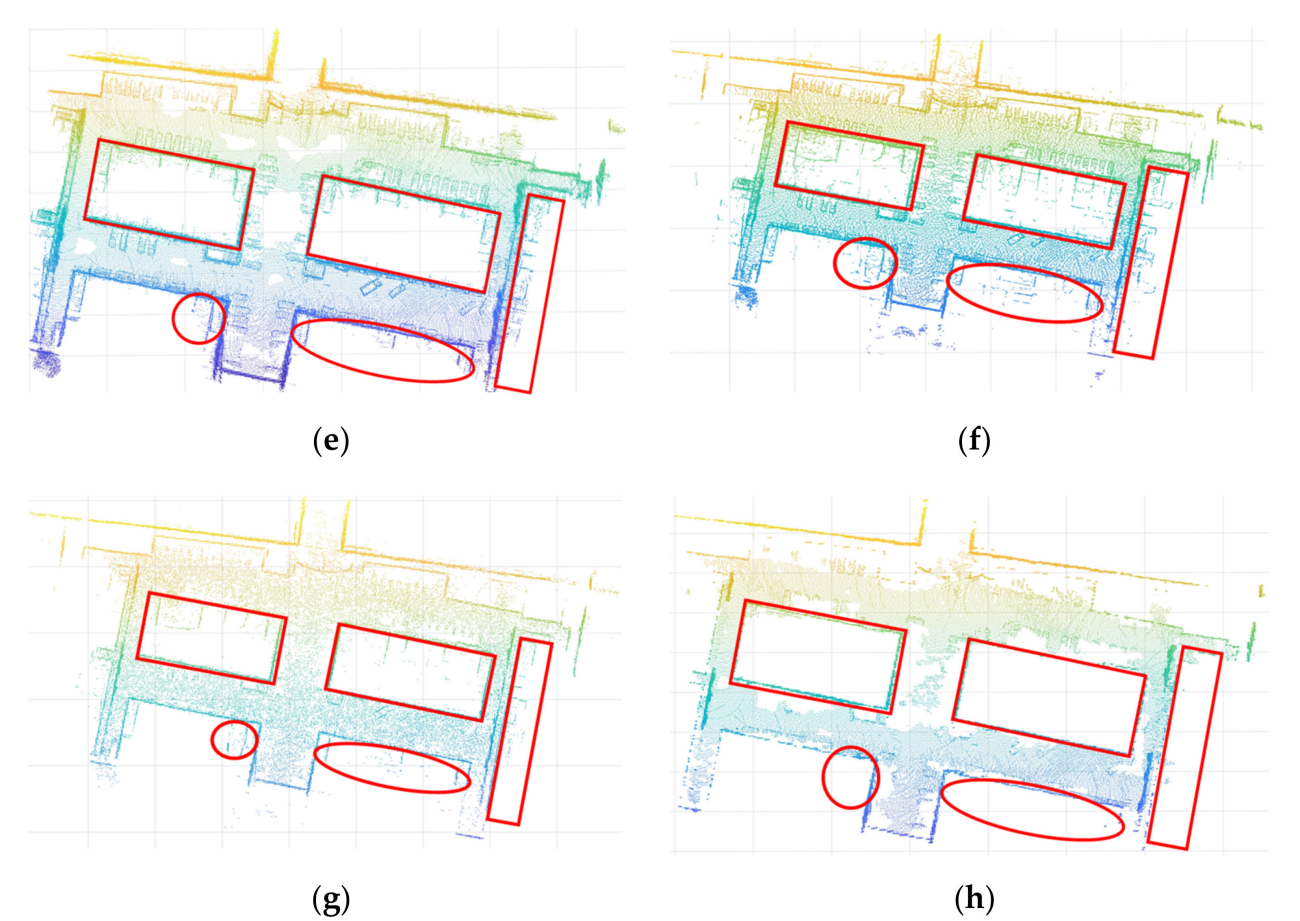
The results of several filtering processes, where the main noise is marked within the red shape: (**a**) the initial data collected; (**b**) the data processed by artificial filtering on the initial data; (**c**) the data processed by the statistical outlier removal (SOR) filter; (**d**) the data processed by the SOR filter on the basis of voxel grid filter processing (the voxel grid statistical outlier removal (VGSOR) filter); (**e**) the data processed by the radius outlier removal (ROR) filter; (**f**) the data processed by the Gaussian filter; (**g**) the data processed by the difference of normals (DON) method; and (**h**) the data processed by the GPCA method.

In order to verify the effectiveness of the algorithm, the proposed algorithm was used to compare the SOR filter, the VGSOR filter, the ROR filter, the Gaussian filter, and the DON filter. The algorithm was evaluated by processing the same data. The raw data are shown in Figure 9a. The main indoor noise was removed manually, as shown in Figure 9b. Noise marks were used to compare the effects of the filters. The results of SOR filtering are shown in Figure 9c. The results of VGSOR filtering are shown in Figure 9d. The result of ROR filtering is shown in Figure 9e. The results of Gaussian filtering are shown in Figure 9f. The results of DON filtering are shown in Figure 9g. The results obtained from this experiment after the data were processed using the GPCA method are shown in Figure 9h. The denoising degree and time complexity are very important to evaluate the performance of filters. We calculated the noise elimination rate and time complexity of these filters, as shown in Table 1. The higher the noise elimination rate and the lower the time complexity, the better the filtering performance. Therefore, it can be seen that the GPCA method has an excellent effect on the processing of edge noise and can effectively remove block noise outside the plane. Based on the ratio of the edge noise removed by the filter to the original noise, the denoising accuracy of GPCA is better than that of the other filters. At the same time, our method also has low time complexity.

**Figure 10:**
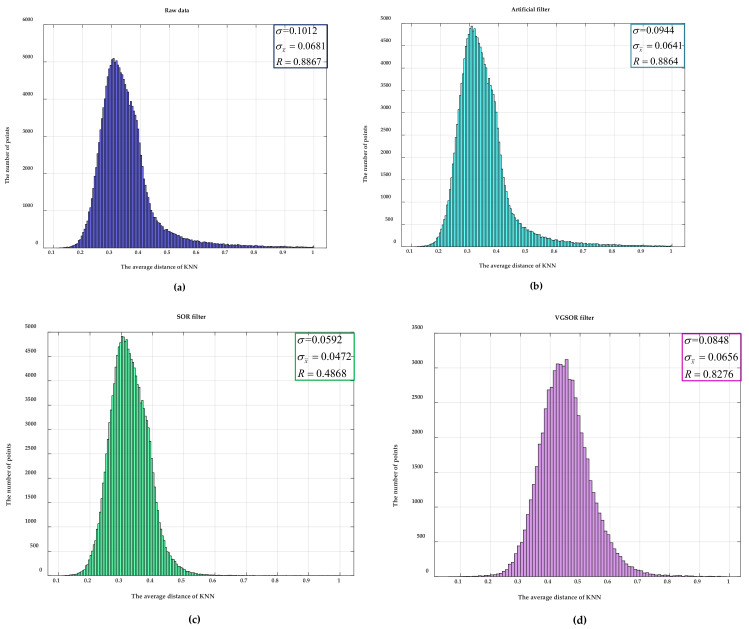

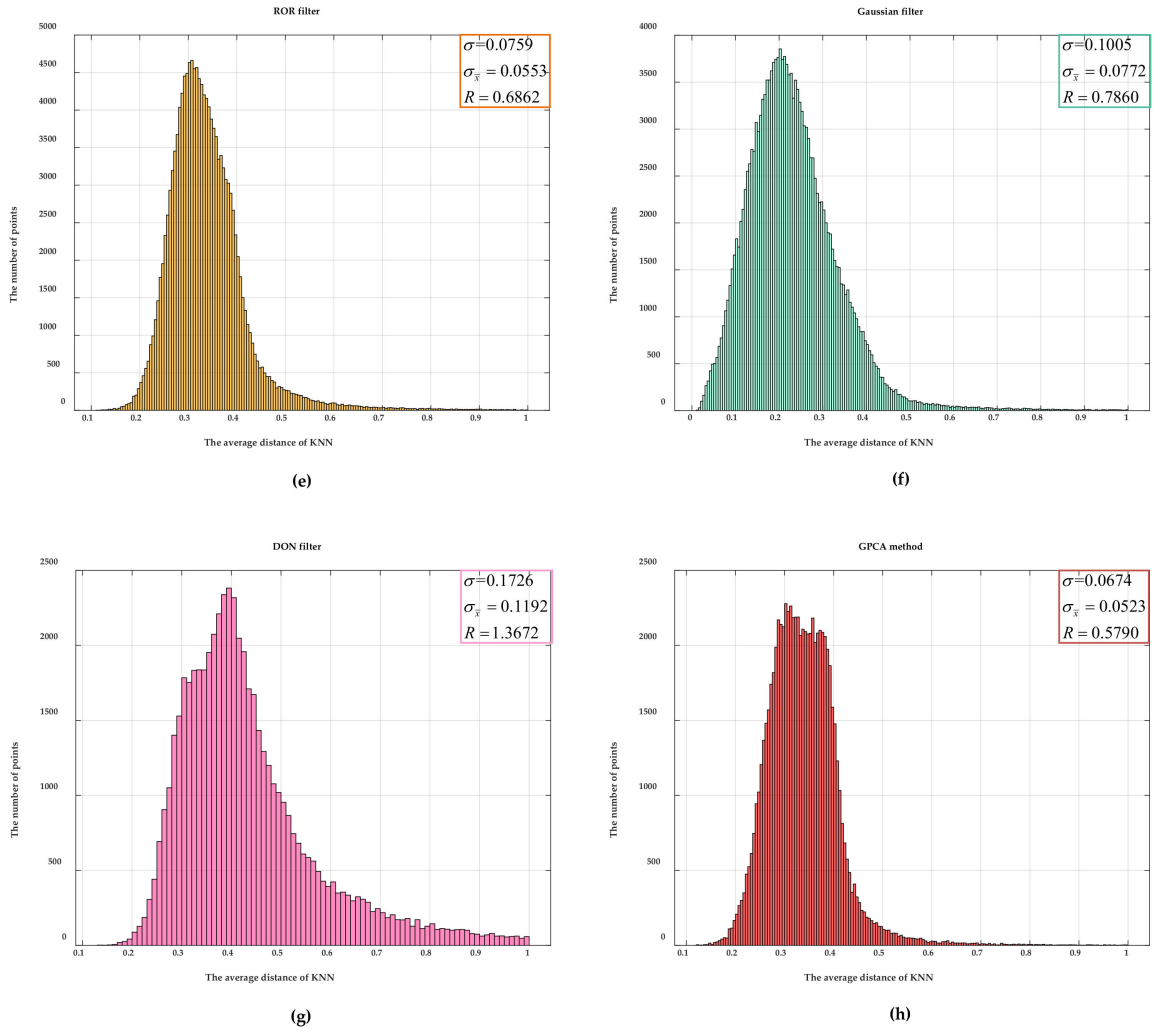
A statistical graph of the average distance of KNN: this average distance is used to evaluate the degree of data dispersion. We calculated three evaluation parameters for the initial and several filtered data. The standard deviation is expressed as σ. The mean variation is expressed as $\sigma_{\bar{x}}$. The range is expressed as R. (**a**) A statistical graph of the raw data, (**b**) a statistical graph of the data filtered by the artificial filter, (**c**) a statistical graph of the data filtered by the SOR filter, (**d**) a statistical graph of the data filtered by the VGSOR filter, (**e**) a statistical graph of the data filtered by the ROR filter, (**f**) a statistical graph of the data filtered by the Gaussian filter, (**g**) a statistical graph of the data filtered by the DON filter, and (**h**) a statistical graph of the data filtered by the GPCA method are presented.

**Figure 11:**
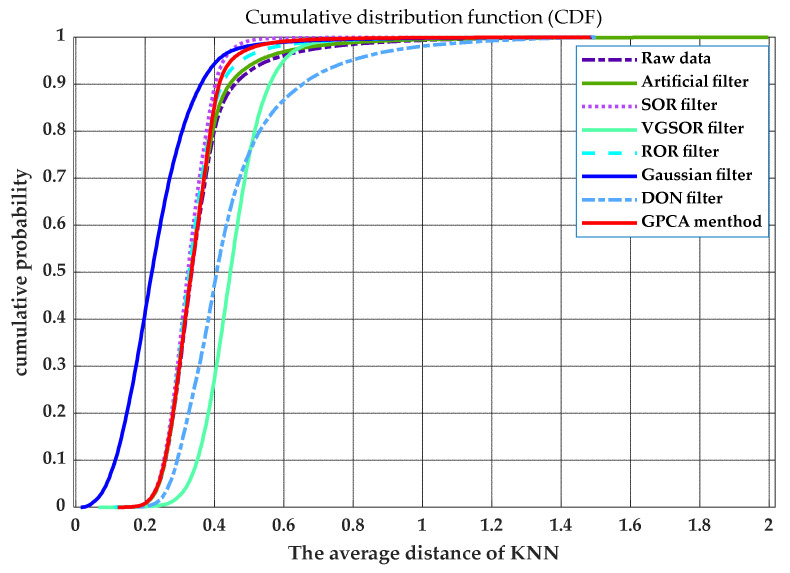
Cumulative distribution function (CDF) of several filters: if F(x) is represented as a CDF, then F(a) is the proportion of points for which the average distance is less than a. The faster the value of cumulative probability increases, the higher the concentration of data. Therefore, it can be used as an indicator to judge the degree of data dispersion.

According to the denoising principle of the SOR filter, the average distance of KNN is used to determine whether it is an outlier. Therefore, the dispersion degree of a point cloud can be judged by the dispersion degree of the average distance of KNN. We calculated the average distance of KNN at each point after filtering, and these mean distances are counted, as shown in Figure 10. In the figure, we calculated the standard deviation σ, mean variation $\sigma_{\bar{x}}$, and range R as shown in Table 1. The smaller the value of these parameters, the lower the degree of dispersion of the data. Therefore, these parameters can be used to evaluate the filter’s performance in removing outliers. Then, the cumulative distribution function of average distance was used to compare each filter intuitively, as shown in Figure 11. According to these parameters, we determined that after the data were processed by our method, the degree of dispersion of the data is slightly lower than the SOR filter and higher than other filters.

## 4. Discussion

In Figure 9, we took the data after manually removing most of the out-of-plane noise as the standard. Through a comparison of several filtering results, we showed that our method is better than other filtering methods at removing noise outside the plane. However, while removing noise, features of objects outside the plane, such as cars and trees, can also be lost. Therefore, our method is suitable for the three-dimensional modeling of buildings. This method can reduce the modeling error caused by a block of trees and other objects in 3D modeling. At the same time, our method also has some advantages in terms of time complexity.

The degree of dispersion of point cloud data can also be used as one of the indicators in evaluating the quality of filtering. Therefore, we used the principle of the SOR filter to obtain the statistics on the average distance of KNN. The dispersion degree of point cloud data after filtering was judged by analyzing the dispersion degree of statistical data. As shown in Figure 10, the statistical data of eight types of data were compared, and the indicators of each type of data were calculated. The smaller the values of the three indicators, the lower the degree of dispersion of the data. Figure 11 is the cumulative probability distribution of the statistical data. When the value of the cumulative distribution function changes from 0 to 1, the faster the abscissa changes, the lower the degree of dispersion of the data. The SOR filter is designed based on the dispersion of data. Therefore, our method is slightly worse than the SOR filter but higher than other filters in terms of the dispersion of the data.

Through an analysis of the degree of data dispersion, as shown in Table 1, it can be seen that our method is slightly lower than the SOR filter in three indicators and higher than other filters. The reason for this is that when the laser radar scans the upper end of the building, the angle between the laser and the plane is too small and, thus, the points there are very sparse. The SOR filter filters it, but our method preserves the point as a plane. If we need to eliminate this point, we simply apply SOR filtering on the basis of our method.

Overall, from the point of view of the denoising degree, time complexity, and discrete degree of processed data, our proposed method is superior to other types of filtering in general.

## 5. Conclusions

The algorithm proposed in this paper can effectively remove noise outside of a plane. The GPCA method was used to accurately extract the vertical plane of the wall and to isolate the noise containing the ground. The NKNN method was used to extract the ground point cloud from the noise obtained in the previous step. Finally, the filtered point cloud was composed of the wall and ground. From the experimental results, it can be seen that the denoising rate of our method is 98%, which is much higher than other filters. Therefore, the proposed method is better than other filters at removing out-of-plane noise and has certain advantages in operational efficiency. Therefore, for the three-dimensional reconstruction of a building surface, our method is a very ideal filter. The disadvantage of this method is that the appropriate parameters need to be adjusted to obtain the best effect; therefore, we will try to improve upon this as adaptive filtering in future work.

## Figures and Tables

**Table 1:** Measurement parameters of several filters.

Filter	Noise Elimination Rate	Time Complexity	σ	$\sigma_{\bar{x}}$	R
SOR filter	53.81%	$O(3n^{2})$	0.0592	0.0472	0.4868
VGSOR filter	87.33%	$O(5n^{2})$	0.0848	0.0656	0.8276
ROR filter	49.31%	O(n)	0.0759	0.0553	0.6862
Gaussian filter	36.25%	$O(n^{2})$	0.1005	0.0772	0.7860
DON filter	80.97%	$O(2n^{2})$	0.1726	0.1192	1.3672
GPCA method	98.03%	$O(n^{2})$	0.0674	0.0523	0.5790

## Data Availability

Not applicable.
